# Supermarket Choice, Shopping Behavior, Socioeconomic Status, and Food Purchases

**DOI:** 10.1016/j.amepre.2015.04.020

**Published:** 2015-12

**Authors:** Rachel Pechey, Pablo Monsivais

**Affiliations:** 1 Behaviour and Health Research Unit, Institute of Public Health, University of Cambridge, Cambridge, United Kingdom;; 2 Centre for Diet and Activity Research, University of Cambridge, School of Clinical Medicine, Institute of Metabolic Science, Cambridge, United Kingdom

## Abstract

**Introduction:**

Both SES and supermarket choice have been associated with diet quality. This study aimed to assess the contributions of supermarket choice and shopping behaviors to the healthfulness of purchases and social patterning in purchases.

**Methods:**

Observational panel data on purchases of fruit and vegetables and less-healthy foods/beverages from 2010 were obtained for 24,879 households, stratified by occupational social class (analyzed in 2014). Households’ supermarket choice was determined by whether they ever visited market-defined high- or low-price supermarkets. Analyses also explored extent of use within supermarket choice groups. Shopping behaviors included trip frequency, trip size, and number of store chains visited.

**Results:**

Households using low-price (and not high-price) supermarkets purchased significantly lower percentages of energy from fruit and vegetables and higher percentages of energy from less-healthy foods/beverages than households using high-price (and not low-price) supermarkets. When controlling for SES and shopping behaviors, the effect of supermarket choice was reduced but remained significant for both fruit and vegetables and less-healthy foods/beverages. The extent of use of low- or high-price supermarkets had limited effects on outcomes. More-frequent trips and fewer small trips were associated with healthier purchasing for both outcomes; visiting more store chains was associated with higher percentages of energy from fruit and vegetables.

**Conclusions:**

Although both supermarket choice and shopping behaviors are associated with healthfulness of purchases, neither appears to contribute to socioeconomic differences. Moreover, differences between supermarket environments may not be primary drivers of the relationship between supermarket choice and healthfulness of purchases.

## Introduction

There are substantial socioeconomic inequalities in the prevalence of non-communicable diseases, key determinants of which are behavioral risk factors, including unhealthy diets.^1^ Unhealthy diets (in particular, eating fewer fruits and vegetables) are also strongly patterned by SES.^2^, ^3^, ^4^, ^5^, ^6^

Neighborhood food environments may drive some of the socioeconomic differences in diet quality. Those who are more deprived may have less physical access to healthier food outlets and greater exposure to unhealthy outlets.^7^, ^8^, ^9^ However, physical proximity to outlets may not adequately represent ability or motivation to use facilities, with a recent U.S. study^10^ suggesting only one third of respondents primarily shopped at their nearest supermarket. Shoppers may also engage in trip chaining or use stores for purchasing different items, diminishing the importance of location.^11^, ^12^ Moreover, U.S. and United Kingdom (UK) studies^13^, ^14^, ^15^, ^16^ suggest that improving physical access to supermarkets may not improve diet quality.

Differences in food costs between supermarkets are another potential driver of supermarket choice, and may contribute to socioeconomic differences in diet quality.^17^ Moreover, consumers who patronized low-priced supermarkets were found to have lower-quality diets^18^ and higher BMI,^19^, ^20^, ^21^ even after adjusting for SES. These differences in diet quality may relate to individual or cultural factors (e.g., prioritizing cost may lead to preferences for certain supermarkets and also limit dietary options within store). Alternatively, or in addition, supermarket environments (e.g., range, price, promotions) may influence purchases,^22^, ^23^, ^24^, ^25^ and this environment may differ systematically between supermarkets in different price tiers.^26^ If differences in supermarket environments are largely driving differences in purchasing by supermarket choice, then a dose–response relationship might be expected (whereby lighter users of low-cost supermarkets have healthier purchases than heavier users). To date, studies have primarily focused on the influence of self-reported primary supermarket, with little evaluation of the impact of the extent of use of different supermarkets on purchasing.

Beyond the type of supermarket patronized, food shopping patterns may affect diet quality. Previous work suggests that shopping behaviors may be associated with differences in purchasing, such as unplanned purchases being more likely during larger shopping trips compared to fill-in trips,^27^ and among shoppers using low-cost stores, but this latter effect was diminished if customers also shopped at multiple stores.^28^

Together, these studies suggest that both supermarket choice and shopping behaviors may shape food purchases, and subsequently the healthfulness of diets. This study aims to explore supermarket choice (including extent of use of different supermarkets) alongside shopping behavior, to provide a more nuanced account of how these factors may impact healthfulness of and social patterning in purchasing.

## Methods

### Study Sample

Data were obtained from the Kantar WorldPanel (KWP) UK household survey from 2010, which comprised purchase records of 26,922 households over 52 weeks (as this involved analyzing de-identified existing data, ethical approval was not required). Households were recruited by KWP via postal mail and e-mail, using quotas determined by the UK Office of National Statistics census and the UK Broadcasters’ Audience Research Board Establishment survey to ensure national representativeness in terms of UK region of residence, age group, and household size. Households received vouchers for high street retailers or leisure activities (approximately equivalent to £100/year) as an incentive toward participation.

Participating households were asked to record all food and beverage purchases brought home. Purchases were recorded using barcode scanners (using showcards with barcodes for non-barcoded products like fruit). Panelists also uploaded digital images of cash register receipts, which were used to verify the accuracy of households’ data. Data included volume purchased, nutritional content, and the retail chain from which products were purchased.

To be included in the data set, households had to meet quality control criteria. These included meeting minimum volume and spending for purchases based on household size, every 4 weeks. In addition, households were included only if they reported at least 12 weeks’ data and at least 13 trips over 52 weeks (equivalent to one trip/4-week period). This gave a final sample of 24,879 households.

### Measures

The KWP data set categorizes head-of-household occupation using the UK Registrar General’s social class classification,^29^ from which we defined three groups: higher managerial and professional (“higher,” *n*=5,332); white collar and skilled manual (“middle,” *n*=13,621); and semi-skilled and unskilled manual (“lower,” *n*=5,926).

We categorized all supermarkets in the KWP data set as high, medium, or low cost based on market definitions, whereby high-cost stores prioritize product quality over price, low-cost stores operate as “discounters,” and those falling between are medium-cost stores.^30^, ^31^ Shopping at convenience stores (accounting for 4% of calories purchased on average) was excluded, given concerns that these trips were more likely to be missing from the data set (see sensitivity analyses section). High-cost (M&S, Ocado, Waitrose), medium-cost (Asda, The Co-operative, Morrisons, Sainsbury’s, Somerfield, Tesco), and low-cost (Aldi, Farmfoods, Iceland, Lidl) supermarket categories included all store sizes for the store chain and online sales.

Households were then classified according to the types of supermarkets they reported visiting. As nearly 100% of households shopped at medium-cost supermarkets over the year (24,828/24,879), supermarket choice groups were determined by whether or not households additionally ever patronized high- or low-cost supermarkets. This gave four groups: used low-cost supermarkets or low- and medium-cost supermarkets (termed “low-cost” for succinctness); used medium-cost supermarkets only (“medium-cost”); used high-cost supermarkets or medium- and high-cost supermarkets (“high-cost”); and used high-, medium-, and low-cost supermarkets (“all-types”).

The extent of use of different types of supermarkets was characterized in terms of the percentage of trips that took place at each supermarket price tier.

Four characteristics of shopping behavior were examined:

1. the number of trips recorded by each household each month;
2. the percentage of small trips (defined as those where ten items or fewer were purchased; see sensitivity analyses section);
3. the mean number of different store chains used per month; and
4. whether or not households used the same primary store chain (i.e., the store chain where they purchase the largest number of items) every month.

We used two outcome variables assessing healthfulness of food and beverage purchases, comprising less-healthy and healthier indices:

1. percentage of energy from less-healthy foods and non-alcoholic beverages, as classified by UK Food Standards Agency Nutrient Profile^32^ scores for individual products (scores are calculated from the energy, saturated fat, sugar, sodium, fiber, protein, and fruit, vegetable, and nut content per 100 g; foods scoring ≥4, and beverages ≥1, are categorized as less healthy); and
2. percentage of energy from fruit and vegetables—this included fresh, canned, frozen, and dried fruit, vegetables, and legumes but excluded juice, potatoes, and fruit and vegetables present in processed products.

### Statistical Analysis

Multiple regression analyses were conducted in 2014 to estimate the percentage of energy purchased from less-healthy foods/beverages and fruit and vegetables by supermarket choice and the four shopping behavior variables. To address the hypotheses relating to social patterning, analyses were conducted with and without controlling for SES (using dummy variables, with the higher group as the ref group).

Analyses (using Stata MP, version 13, “regress” command) used robust SEs, given evidence of heteroscedasticity, and the percentage of energy from fruit and vegetables was logged to address a positively skewed distribution. Reported significance levels were adjusted for multiple testing using Bonferroni correction.

Analyses were also conducted within supermarket choice groups to examine whether extent of use of different supermarket tiers affects healthfulness of purchases (over and above being identified as an “ever shopper” at these store chains).

Regressions controlled for age of main shopper, gender of main shopper, ethnic group of main shopper (white/non-white); number of adults in household, number of children in household, and dummies for region of residence (Midlands, North East, Yorkshire, Lancashire, South, Scotland, Anglia, Wales & West, South West). All households meeting study eligibility criteria had complete demographic data, so no households were excluded because of missing values.

Sensitivity analyses were conducted (1) using a different cut off threshold for small trips (20 items) and (2) including trips to convenience stores. Both these sets of analyses found very similar results to those in the main analyses, so these results are not included here.

## Results

The descriptive data presented in Table 1 indicated social patterning associated with supermarket choice. Among the low-cost supermarket users, 15% were from the highest occupational social class group and 31% were from the lowest group. By contrast, 36% of the higher versus 12% in the lower occupational social class group shopped in high-cost supermarkets. Similar patterning was seen across income groups.

Table 1 also suggests that supermarket choice may be associated with healthfulness of purchases, with 1.2–1.5 percentage point differences for the mean energy from less-healthy foods/beverages and from fruit and vegetables between the low- and high-cost supermarket groups.

However, the supermarket choice groups may also show somewhat different patterns of shopping behavior: the low-cost supermarket group had on average a slightly higher number of trips per month and visited more different store chains than the high-cost supermarket group (although there are more low-cost [*n*=4] than high-cost [*n*=3] store chains).

Table 2 shows results for the percentage of energy households obtained from less-healthy foods/beverages. Although analyses only including supermarket choice suggested the low-cost supermarket group purchased significantly higher percentages of energy from less-healthy foods/beverages than the high-cost supermarket group (by 1.3 percentage points), these differences were reduced (to 0.77 percentage points) when controlling for SES. Additionally controlling for shopping behavior had little impact on the difference between the high- and low-cost supermarket groups (–0.77 to –0.72).

Shopping behaviors were significantly associated with healthfulness of purchases, with a 0.9 percentage point decrease in calories from less-healthy foods/beverages for a household at the 75th percentile for trips per month (9.2 trips/month) compared with one at the 25th percentile (3.9 trips/month), whereas a 10 percentage point increase in the percentage of small trips per month was associated with a 0.4 percentage point increase in the percentage of calories obtained from less-healthy foods/beverages. Including shopping behaviors had little effect on the coefficients for SES.

Table 3 shows the results for the percentage of energy obtained from fruit and vegetables. As this outcome was logged to address a skewed distribution, Table 3 presents the percentage change per unit change in predictors (calculated from back-transformed [exponentiated] *b* coefficients). The high-cost supermarket group purchased 9% higher percentages of energy from fruit and vegetables on average compared with the low-cost supermarket group, when controlling for shopping behaviors and SES. The coefficients for supermarket choice, shopping behavior, and SES were largely similar when controlling for one another.

In terms of shopping behavior, results suggest a 3% increase in the percentage of calories from fruit and vegetables for households at the 75th compared with the 25th percentile for trips per month, whereas for a 10 percentage point increase in the percentage of small trips per month, the percentage of calories from fruit and vegetables decreased by 6%. For each additional store chain visited per month, results suggest the percentage of calories households obtain from fruit and vegetables increased by 6%.

The above analyses examined the effect of ever shopping at supermarkets in different price tiers (meaning there was considerable variability in the extent of use of each supermarket price tier within groups). Table 4 shows the results of analyses addressing the extent of use of low- and high-cost supermarkets within the low-cost, high-cost, and all-types supermarket choice groups. These suggest that for the low-cost supermarket group, using low-cost supermarkets for a greater percentage of trips was not associated with the percentage of calories obtained for fruit and vegetables, but it was associated with a small reduction in the percentage of calories purchased from less-healthy foods/beverages (0.25 percentage point decrease for a 10 percentage point increase in percentage of trips at low-cost supermarkets). The effects of extent of use of low-cost supermarkets were similar for the all-types supermarket group.

For the high-cost supermarket group, there was no association between percentage of trips at high-cost supermarkets and percentage of calories for less-healthy foods/beverages. The effects of extent of use of high-cost supermarkets were again similar for the all-types supermarket group. A greater percentage of trips at high-cost supermarkets was associated with a greater percentage of calories being obtained from fruit and vegetables, but suggested a small effect: For the high-cost supermarket group, for a 10 percentage point increase in the percentage of trips at high-cost supermarkets, the percentage of calories from fruit and vegetables increased by 2%. The social patterning in purchasing was reduced within the high-cost supermarket group.

## Discussion

Our analyses of a large UK data set indicate that supermarket choice might have implications for diet quality and health. Compared with households that ever used lower-cost supermarkets, households that ever used high-cost supermarkets purchased 9% higher percentages of energy from fruits and vegetables and 0.8 percentage points less energy from less-healthy foods/beverages. At the population level and over time, such differences in fruit and vegetable purchasing may be clinically significant. These results are consistent with previous studies, which found that consumers who patronized low-cost supermarkets consumed less nutritious diets and fewer vegetables and fruit.^10^, ^18^

Comparing the “ever use” analyses to the “extent of use” analyses—showing smaller (sometimes counterintuitive) or non-significant effects of supermarket use—suggested the latter had limited effects on purchasing. The lack of a dose–response relationship here suggests that differences in supermarket environments (e.g., the range of fruit and vegetables available, use of in-store marketing) may not be contributing greatly to differences in purchasing by supermarket price tier. Although we did not directly examine individual or cultural factors beyond SES, this may suggest that such factors (that may prompt individuals to choose a particular type of supermarket) may be more-influential drivers of disparities in the healthfulness of purchases by supermarket price tier. Differences largely were not affected by socioeconomic adjustment, however, suggesting that supermarket choice and SES have independent effects on purchasing behavior, consistent with previous U.S. results.^10^

Unique to this study was the assessment of shopping behavior in the context of supermarket choice and SES. Though the measures of shopping behavior available in this data set were relatively limited, constraining the potential for comparisons with previous research, more shopping trips per month and a smaller percentage of small trips in particular were associated with more healthful food patterns. The inclusion of shopping behaviors in these analyses provided initial evidence that these variables were not confounding the association between supermarket choice and healthfulness of purchasing. In addition, including shopping behavior variables did not alter SES coefficients, suggesting these do not contribute to the socioeconomic differences in purchasing. This ties in with previous work^33^ suggesting that a range of shopping patterns are adopted within different socioeconomic groups. As such, changing patterns of shopping behavior may have the potential to improve diet quality for shoppers across the socioeconomic spectrum, regardless of supermarket choice. Further research is needed here, however, to address causality. For example, smaller “fill-in” trips may be associated with greater time pressure, which may make less-healthy food choices more likely.^34^ Alternatively, those who purchase more fruit and vegetables may choose to undertake more-frequent shopping trips to obtain these perishable items.

### Limitations and Methodological Considerations

There are several limitations that need to be noted. First, these results reflect purchasing and as such may not translate directly to diet. In addition, we used market definitions to determine supermarket price tiers, which may reflect perceptions of store differences rather than, or in addition to, differences in price or other store characteristics. Regarding the representativeness of the data set, the overall low recorded volumes of food and beverages suggest under-reporting (with households reporting on average approximately three quarters of the in-home calories, excluding alcohol, reported by households in the Family Food survey from 2010),^35^ and smaller shopping trips may be more likely to be missed when reporting data. As such, the coefficient for percentage of smaller trips in particular should be treated with caution, as it is possible this reflects in part households’ willingness to report even their smaller trips. In addition, the types of purchased foods may differ in these smaller trips; for example, fruit and vegetables may be more likely to be purchased in “fill-in” trips. Sensitivity analyses using a different threshold for defining small trips and including trips to convenience stores found similar results, however. Moreover, examining the degree of under-reporting suggested that this did not vary systematically by SES; comparisons of under-reporting by income quintiles suggested that households in the KWP data set in the second and fourth income quintiles reported around 68%–69% of the calories reported by the same quintiles in the Family Food survey in 2010, increasing to between 73% and 80% for the first, third, and fifth quintiles.^2^, ^35^

Nonetheless, this study offers a more robust account of the association between supermarket choice and food/beverage purchases than previous work, by employing detailed scanner data. In addition, the novel inclusion of shopping behavior variables provides initial evidence as to their potential to influence healthfulness of purchases. Investigating indices of both healthier and less-healthy purchasing, the drivers of which may be different,^36^ provides a more nuanced account of the relationships between supermarket choice and shopping behaviors and the healthfulness of purchasing.

### Implications for Research and Policy

Further research investigating the way in which households conduct their shopping would be beneficial, to establish whether encouraging certain patterns of shopping behavior may help to promote healthier purchases. However, the present results (whereby including shopping behavior and supermarket price tier in analyses had very little impact on SES coefficients) suggest that addressing supermarket choice or shopping behavior is unlikely to substantially impact socioeconomic differences in healthfulness of purchases, and other public health measures may need to be considered to target these inequalities.

### Conclusions

Supermarket choice is associated with small differences in the healthfulness of purchases. However, differences between supermarket environments may not be primary drivers of this association—that is, high-cost supermarkets may not be differentially encouraging healthier purchasing nor low-cost supermarkets differentially encouraging less-healthy purchasing.

## Figures and Tables

**Table 1:** Household and Main Shopper Characteristics by Supermarket Choice Group

**Characteristic**	**Supermarket choice group**			
**Low-cost**	**Medium-cost**	**High-cost**	**All-types**
*n*	9,161	5,111	3,183	7,414
Percentage female	78.2%	77.7%	77.5%	79.5%
Age, M (SD)	48.3 (15.1)	44.3 (14.9)	50.1 (16.1)	54.5 (14.9)
Percentage white	94.6%	95.4%	95.5%	96.0%
Number of adults in household, M (SD)	2.10 (0.90)	2.05 (0.85)	1.95 (0.80)	2.06 (0.84)
Number of children in household, M (SD)	0.74 (1.06)	0.81 (1.08)	0.49 (0.84)	0.42 (0.83)
Occupational social class,^a^ %				
Higher	15.3	22.3	35.8	22.2
Middle	53.8	56.3	52.3	55.9
Lower	30.9	21.4	11.9	21.8
Equivalized income (£/year), %				
Missing	23.6	23.4	25.3	24.1
0–9,999	23.0	16.6	8.3	14.8
10,000–19,999	36.4	32.3	27.0	36.2
20,000–29,999	10.7	14.2	16.9	13.5
≥30,000	6.3	13.6	22.5	11.5
Number of trips per month, M (SD)	7.16 (4.78)	4.19 (2.58)	6.42(3.96)	9.65 (5.47)
Percentage of trips that are small (≤10 items)	43.8% (25.1)	24.1% (24.8)	40.6% (24.5)	54.2% (21.8)
Number of different store chains per month, M (SD)	2.66 (1.04)	1.53 (0.54)	2.27 (0.80)	3.50 (1.23)
Percentage using same primary store chain every month, M (SD)	32.9%	56.0%	40.3%	29.0%
Percentage of trips to low-cost stores, M (SD)	20.8% (19.5)	—	—	16.7% (16.4)
Percentage of trips to medium-cost stores, M (SD)	79.2% (19.5)	100% (n/a)	82.5% (22.3)	74.1% (19.4)
Percentage of trips to high-cost stores, M (SD)	—	—	17.5% (22.3)	9.2% (13.1)
Percentage of purchased calories from less-healthy foods/ beverages, M (SD)^b^	52.0% (9.7)	51.2% (10.2)	50.5% (9.8)	51.3% (9.4)
Percentage of purchased calories from fruit and vegetables^c^	6.57% (3.91)	6.96% (4.16)	7.78% (4.50)	7.26% (4.11)

a Occupational social class: “higher,” higher managerial and professional; “middle,” white collar and skilled manual; “lower,” semi-skilled and unskilled manual.

b Less-healthy foods and beverages were defined by Food Standards Agency Nutrient Profile (28) scores for individual products (foods scoring ≥4, and beverages ≥1).

c Fruit and vegetables included fresh, canned, frozen and dried fruit, vegetables and legumes, but excluded juice, potatoes, and fruit and vegetables present in processed products.

**Table 2:** Estimated Percentage of Calories From Less-Healthy Foods/Beverages

	Supermarket choice only	SES only	Supermarket choice and SES	Shopping behavior only	Shopping behavior and SES	All variables
Supermarket choice (ref group: Low-cost)						
Medium-cost	–0.54 (–1.13, 0.05)		–0.33 (–0.92, 0.26)			–0.13 (–0.77, 0.50)
High-cost	–1.32^***^ (–2.00, –0.64)		–0.77^*^ (–1.46, –0.07)			–0.72^*^ (–1.41, –0.02)
All-types	–0.78^***^ (–1.29, –0.28)		–0.50 (–1.01, 0.00)			–0.34 (–0.87, 0.19)
SES (occupational social class; ref group: Higher)						
Middle		1.06^***^ (0.54, 1.58)	0.99^***^ (0.46, 1.51)		1.04^***^ (0.52, 1.56)	0.98^***^ (0.45, 1.50)
Lower		2.92^***^ (2.30, 3.55)	2.78^***^ (2.15, 3.42)		2.88^***^ (2.26, 3.50)	2.75^***^ (2.12, 3.39)
Shopping behavior						
Number of trips per month^a^				–0.88^***^ (–1.26, –0.50)	–0.93^***^ (–1.31, –0.55)	–0.92^***^ (–1.30, –0.54)
Percentage of trips with ≤10 items purchased^b^				0.43^***^ (0.31, 0.56)	0.40^***^ (0.28, 0.53)	0.40^***^ (0.28, 0.53)
Number of store chains visited per month				–0.24 (–0.51, 0.04)	–0.21 (–0.48, 0.07)	–0.19 (–0.49, 0.11)
Same primary store chain used each month (ref group: No)				–0.27 (–0.75, 0.21)	–0.29 (–0.77, 0.18)	–0.29 (–0.76, 0.19)

*Note:* Values are B coefficients, with Bonferroni-corrected 95% CIs in parentheses. Boldface indicates statistical significance (*p<0.05; **p<0.01; ***p<0.001). All CI estimates were Bonferroni corrected for multiple comparisons. Regressions controlled for age of main shopper, gender of main shopper, ethnic group of main shopper, number of adults in household, number of children in household, and region of residence. Low-cost supermarkets and Higher occupational social class are reference groups. Less-healthy foods and beverages were defined by Food Standards Agency Nutrient Profile (28) scores for individual products (foods scoring ≥4, and beverages ≥1).

^a^Scaled to represent the difference between a household at the 25th percentile and one at the 75th percentile (i.e., IQR-scaled).

^b^Scaled to represent a 10 percentage point change.

**Table 3:** Estimated Percentage of Calories From Fruit and Vegetables

	Supermarket choice only	SES only	Supermarket choice and SES	Shopping behavior only	Shopping behavior and SES	All variables
Supermarket choice (ref group: Low-cost)						
Medium cost	5%^***^ (2, 9)		4%^**^ (1, 7)			0% (–3, 3)
High-cost	13%^***^ (9, 17)		9%^***^ (6, 13)			9%^***^ (5, 13)
All-types	7%^***^ (4, 10)		5%^***^ (2, 8)			5%^***^ (2, 7)
SES (occupational social class; ref group: Higher)						
Middle		–9%^***^ (–12, –6)	–8%^***^ (–11, –5)		–7%^***^ (–11, –5)	–8%^***^ (–10, –4)
Lower		–20%^***^ (–25, –18)	–19%^***^ (–23, –15)		–19%^***^ (–22, –15)	–16%^***^ (–20, –14)
Shopping behavior						
Number of trips per month^a^				3%^***^ (1, 5)	3%^***^ (1, 5)	3%^***^ (1, 5)
Percentage of trips with ≤10 items purchased^b^				–6%^***^ (–6, –5)	–6%^***^ (–6, –5)	–6%^***^ (–6, –5)
Number of store chains visited per month				6%^***^ (5, 8)	6%^***^ (5, 7)	6%^***^ (4, 7)
Same primary store chain used each month (ref group: No)				0% (–3, 2)	0% (–2, 2)	0% (–2, 2)

*Note:* Values are percentage change per unit change in predictor variable, Bonferroni-corrected 95% CIs are in parentheses. Boldface indicates statistical significance (*p<0.05; **p<0.01; ***p<0.001). Percentage change per unit change in predictor variable was determined by back-transforming (exponentiating) B coefficients, and expressing as percentage change. All CI estimates were Bonferroni corrected for multiple comparisons. Regressions controlled for age of main shopper, gender of main shopper, ethnic group of main shopper, number of adults in household, number of children in household, and region of residence. Low-cost supermarkets and Higher occupational social class are reference groups. Percentage of calories from fruit and vegetables includes fresh, canned, frozen and dried fruit, vegetables and legumes, but excludes juice, potatoes, and fruit and vegetables present in processed products.

a Scaled to represent the difference between a household at the 25th percentile and one at the 75th percentile (i.e., IQR-scaled).

b Scaled to represent a 10 percentage point change.

**Table 4:** Estimated Purchasing From Extent of Use of High/Low-Cost Supermarkets Within Supermarket Choice Groups

	Low-cost supermarket group		High-cost supermarket group		All-types supermarket group	
			FV		FV
LHFB B coefficient	FV Percentage change	LHFB B coefficient	Percentage change	LHFBB coefficient	Percentage change
Supermarket use						
Percentage of trips to low-cost supermarkets^a^	–0.25^**^ (–0.46, –0.05)	0% (–1, 9)	—	—	–0.42^***^ (–0.68, –0.17)	1% (0, 2)
Percentage of trips to high-cost supermarkets^a^	—	—	–0.21 (–0.49, 0.08)	2%^***^ (1, 3)	–0.19 (–0.50, 0.11)	2%^*^ (2, 3)
Shopping behavior						
Number of trips per month^b^	–0.60 (–1.21, 0.01)	2% (–1, 5)	–1.76^***^ (–2.94, –0.59)	8%^*^ (1, 15)	–1.12^***^ (–1.71, –0.53)	3%^**^ (0, 7)
Percentage of trips with ≤10 items purchased^a^	0.29^***^ (0.09, 0.49)	–6%^***^ (–8, –5)	0.60^***^ (0.26, 0.94)	–6%^***^ (–9, –5)	0.49^***^ (0.25, 0.74)	–6%^***^ (–9, –5)
Number of store chains visited per month	–0.24 (–0.75, 0.26)	8%^***^ (5, 10)	0.35 (–0.65, 1.35)	4% (–1, 10)	0.03 (–0.42, 0.48)	4%^***^ (2, 6)
Same primary store chain used each month (ref group: No)	–0.62 (–1.40, 0.17)	0% (–4, 4)	0.07 (–1.29, 1.43)	3% (–4, 11)	–0.56 (–1.41, 0.30)	2% (–3, 6)
SES (occupational social class; ref group: Higher)						
Middle	1.07^*^ (0.08, 2.05)	–9%^***^ (–14, –4)	0.17 (–1.07, 1.41)	–3% (–10, 3)	0.99^*^ (0.08, 1.90)	–6%^***^ (–11, –1)
Lower	3.01^***^ (1.93, 4.09)	–19%^***^ (–25, –12)	2.39^**^ (0.30, 4.48)	–15%^***^ (–27, –3)	2.45^***^ (1.32, 3.58)	–12%^***^ (–19, –6)

*Note:* B coefficients for analyses of less-healthy foods/beverages (LHFB); percentage change per unit change in predictor variable for analyses of fruit and vegetables (FV); Bonferroni-corrected 95% CIs are in parentheses. Boldface indicates statistical significance (*p<0.05; **p<0.01; ***p<0.001). Coefficients for fruit and vegetables represent percentage change per unit change in predictor variable, determined by back-transforming (exponentiating) B coefficients, and expressing as percentage change. All CI estimates were Bonferroni corrected for multiple comparisons. Regressions controlled for age of main shopper, gender of main shopper, ethnic group of main shopper, number of adults in household, number of children in household, and region of residence. Higher occupational social class is the reference group for SES.

FV, percentage of calories from fruit and vegetables, including fresh, canned, frozen and dried fruit, vegetables and legumes, but excluding juice, potatoes, and fruit and vegetables present in processed products; LHFB, percentage of calories from less healthy foods and beverages, where less healthy foods and beverages are defined by Food Standards Agency Nutrient Profile (28) scores for individual products (foods scoring ≥4, and beverages ≥1).

a Scaled to represent a 10 percentage point change.

b Scaled to represent the difference between a household at the 25th percentile and one at the 75th percentile (i.e., IQR-scaled).
